# Fluorescently labeled circular DNA molecules for DNA topology and topoisomerases

**DOI:** 10.1038/srep36006

**Published:** 2016-10-31

**Authors:** Maxwell Gu, Andrea Berrido, Walter G. Gonzalez, Jaroslava Miksovska, Jeremy W. Chambers, Fenfei Leng

**Affiliations:** 1 Biomolecular Science Institute, Florida International University, Miami, FL 33199; 2 Department of Chemistry & Biochemistry, Florida International University, Miami, FL 33199; 3 Department of Cellular Biology and Pharmacology, Herbert Wertheim College of Medicine, Florida International University, Miami, FL 33199.

## Abstract

DNA topology plays essential roles in several fundamental biological processes, such as DNA replication, recombination, and transcription. Typically agarose gel electrophoresis is employed to study DNA topology. Since gel electrophoresis is time-consuming and labor intensive, it is desirable to develop other methods, such as fluorescence-based methods, for such studies. In this paper we report the synthesis of a type of unique fluorescence-labeled DNA molecules that can be used to study DNA topology and topoisomerases by fluorescence resonance energy transfer (FRET). Specifically, we inserted an 82 nt. synthetic DNA oligomer FL905 carrying a 42 nt. AT sequence with fluorescein and dabcyl labels into a gapped DNA molecule to generate relaxed and supercoiled pAB1_FL905. Since the fluorescence intensity of pAB1_FL905 is dependent on its supercoiling status, pAB1_FL905 is a powerful tool to study DNA topology and topoisomerases by FRET. pAB1_FL905 can also be developed into rapid and efficient high-throughput screening assays to identify inhibitors that target various DNA topoisomerases.

DNA supercoiling is a fundamental property of chromosomal DNA and plays critical roles in many essential DNA transactions, such as transcription, DNA replication, and recombination^1^,^2^. Usually agarose gel electrophoresis is used to study DNA supercoiling. Since gel electrophoresis is time-consuming and labor intensive, it is desirable to develop other assays, such as fluorescence-based assays, to study DNA topology and topoisomerases. For example, fluorescence dyes, such as PicoGreen^3^, have been shown to differentially bind to supercoiled (sc) and relaxed (rx) DNA molecules to yield different fluorescence properties. These fluorescence dyes were used to study DNA topoisomerases. However, the difference of the fluorescence intensity of the dyes binding to sc and rx DNA is too small to be widely used to study properties of DNA topoisomerases and to screen inhibitors against these topoisomerases^3^.

Another type of assays were developed from the utility of a unique property of sc DNA molecules that prefer binding to triplex-form oligomers if the sc plasmids contain one or multiple triplex-forming sequences^4^,^5^. Maxwell and coworkers invented a method in which an immobilized triplex-forming oligomer more efficiently captures sc plasmids than rx plasmids^4^. The captured plasmids can be subsequently quantified by a DNA-binding dye, such as SYBR Green. However, this method requires immobilization of oligomer to a solid surface, filtration, and multiple washing steps. Since streptavidin-coated 1536-well plates are not commercially available, this method is not compatible with ultra-high throughput screening to identify gyrase inhibitors from small compound libraries using 1536-well plates. Another method, also based on the triplex-forming oligomers, was developed by using fluorescence anisotropy for the readout^5^. Nevertheless, the signal to noise ratio is a concern and an expensive fluorimeter with the capacity to measure fluorescence anisotropy is required^5^.

More recently, Berger and coworkers made a circular plasmid DNA template that contains a fluorophore (fluorescein) and quencher (dabcyl), and developed a real-time assay to study DNA topological changes with this fluorescently labeled DNA^6^. However, the production yield of the fluorescently labeled DNA was too low to allow the assay to be widely used^6^. Additionally, because of the low yield of the DNA substrate, it makes the assay too costly. Here we describe a method to produce a type of fluorescently labeled circular DNA molecules with high yields to study DNA topology and topoisomerases by fluorescence resonance energy transfer (FRET). We also demonstrate that these unique DNA molecules can be used to screen anti-cancer drugs and antibiotics targeting DNA topoisomerases.

## Results and Discussion

### Experimental strategies to construct relaxed (rx) and supercoiled (sc) pAB1_FL905

**Figure 1:**
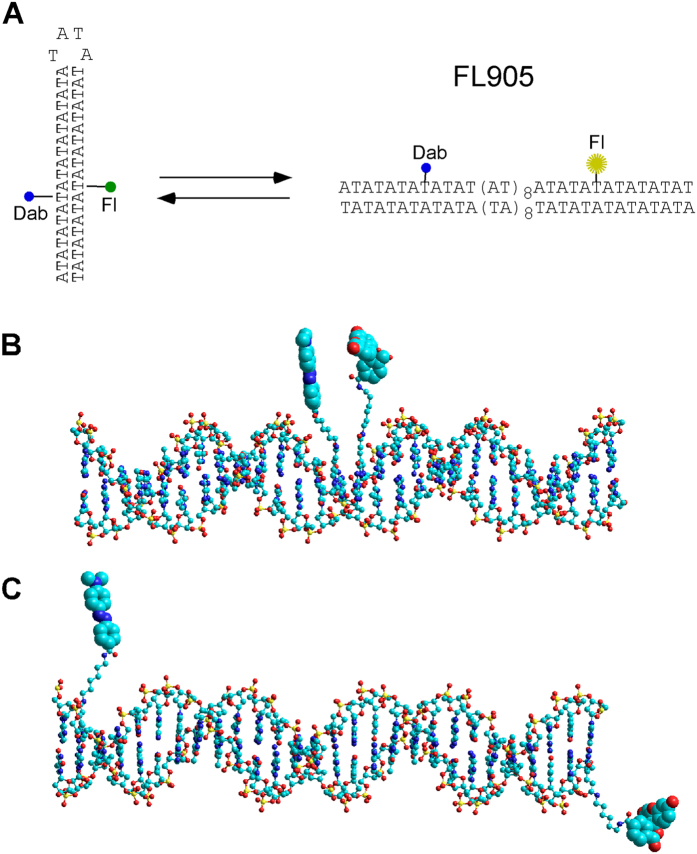
Molecular models of (AT)_42_ DNA carrying fluorescein (Fl) and dabcyl (Dab) labels. (**A**) The 42 nt AT sequence of FL905 can convert from a hairpin structure to an open structure. The fluorescence of Fl is quenched by Dab in the hairpin structure. (**B**) The Fl and Dab labels are in proximity to each other when the (AT)_42_ of FL905 adopts the hairpin structure. (**C**) The positions of the Fl and Dab labels are far away when the (AT)_42_ sequence is in the double stranded state.

As demonstrated previously^7^,^8^,^9^, (-) supercoiling induces localized DNA conformation transitions, such as cruciform formation of inverted repeat sequences. These topology-dependent, structural isomerizations could be used to gauge the superhelicity of the DNA molecules. As shown by Lilley *et al*.^10^ and Mirkin *et al*.^11^,^12^,^13^,^14^, alternating adenine-thymine sequences [(AT)_n_] undergo very rapid cruciform formation, as no detectable kinetic barrier prevents rapid interconversion between extruded and unextruded conformations in sc plasmid DNA templates^10^. We therefore decided to utilize this property of [(AT)_n_] to monitor supercoiling change of plasmid DNA templates. We designed an 82 nt DNA oligomer FL905 that contains a dabcyl-labeled dT at 29th position from the 5′-end (the 8^th^ position of the AT sequence from the 5′ end; Fig. 1A) and a fluorescein-labeled dT at 55th position from the 5′-end (the 34^th^ position of the AT sequence from the 5′ end, Fig. 1A). Figure S1A shows that FL905 has intrinsic fluorescence before EB staining. We reasoned that if the 42 nt AT sequence adopts the hairpin structure, both the fluorescein and dabcyl are in close proximity in the major groove (~20 Å; Fig. 1B). The fluorescence of fluorescein should be greatly quenched. In contrast, when the 42 nt AT sequence adopts the double-stranded DNA form, the distance between the fluorescein and dabcyl should be more than 88.4 Å for B-form DNA (26 bp × 3.4 Å = 88.4 Å). Indeed, our molecular model shows that the distance between the fluorescein and dabcyl is ~100 Å (Fig. 1C). The fluorescence of fluorescein should not be quenched. Figure S1B shows a fluorescence melting experiment in which a four-fold fluorescence intensity change of fluorescein was observed upon the 42 nt AT hairpin structure was melted. This result demonstrates that fluorescence resonance energy transfer (FRET)^15^ can be used to study the interconversion between extruded and unextruded conformations of FL905.

**Figure 2:**
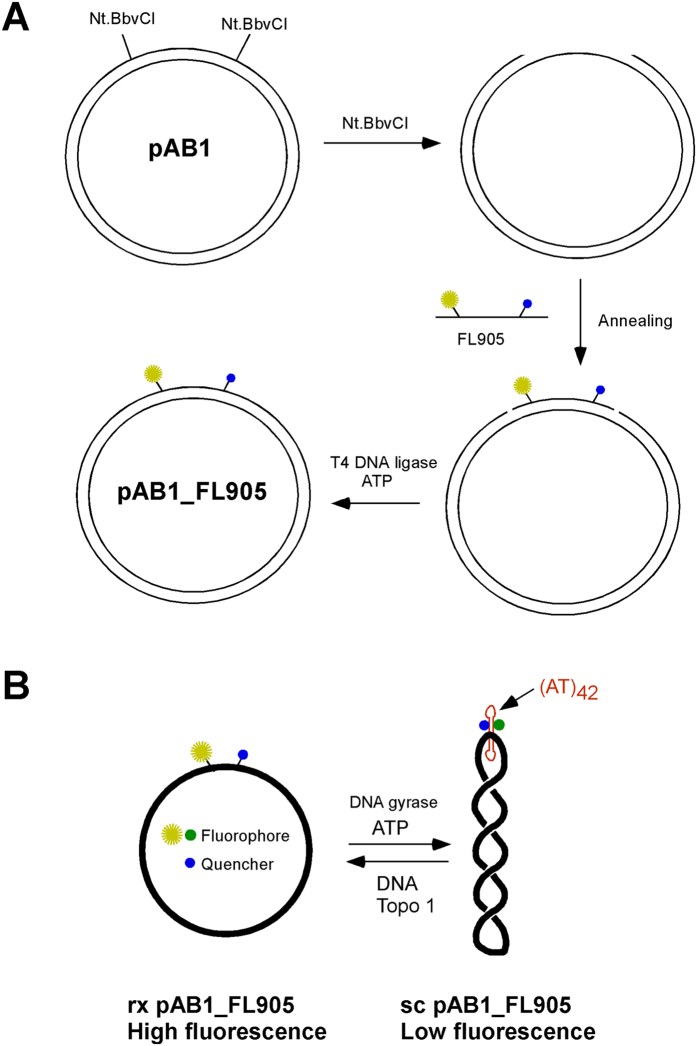
An experimental strategy to construct relaxed (rx) or supercoiled (sc) pAB1_FL905. (**A**) Oligomer FL905 that contains the 42 nt. AT sequence is ligated between the two Nt.BbvCI sites of plasmid pAB1 to yield rx pAB1_FL905. (**B**) Sc pAB1_FL905 can be generated through the treatment of rx pAB1_FL905 by *E. coli* DNA gyrase. The fluorescence intensity of fluorescein is dependent on the supercoiling status of pAB1_FL905.

Next, we constructed pAB1 by inserting a synthetic double-stranded oligomer FL_AT42, which carries the 42 bp AT sequence (Figure S1C), between the SphI and BamHI sites of pUC18 (Figure S1D). Plasmid pAB1 also contains two nicking edonuclease Nt.BbvCI recongnition sites in the same orientation. In this way, DNA oligomer FL905 can be inserted between the two Nt.BbvCI sites according to a strategy described in Fig. 2A (a similar strategy was used to study DNA recombination by Levene and coworks^16^ and label large DNA fragments^17^,^18^). Because rx pAB1_FL905 is the only theoretical ligation product, the production yeild should be near 100%. Sc pAB1_FL905 can be generated through the treatment of rx pAB1_FL905 by bacterial DNA gyrase in the presence of ATP (Fig. 2B). Rx and sc pAB1_FL905 should be powerful tools to study DNA topology and topoisomerases by FRET.

### Fluorescence properties of relaxed, nicked, supercoiled pAB1_FL905

To date, we have produced ~0.5 mg of rx pAB1_FL905 and ~0.6 mg of sc pAB1_FL905 using the strategy described in Fig. 2 and purified by CsCl-EB equilibrium gradient banding with approximately 60% yield. A detailed procedure was provided in “Materails and Methods” and also in Figure S2. Figure S3 shows gel images at various stages of the procedure. As expected, rx and sc pAB1_FL905 have intrinsic fluorescence before EB staining (Figure S3D).

**Figure 3:**
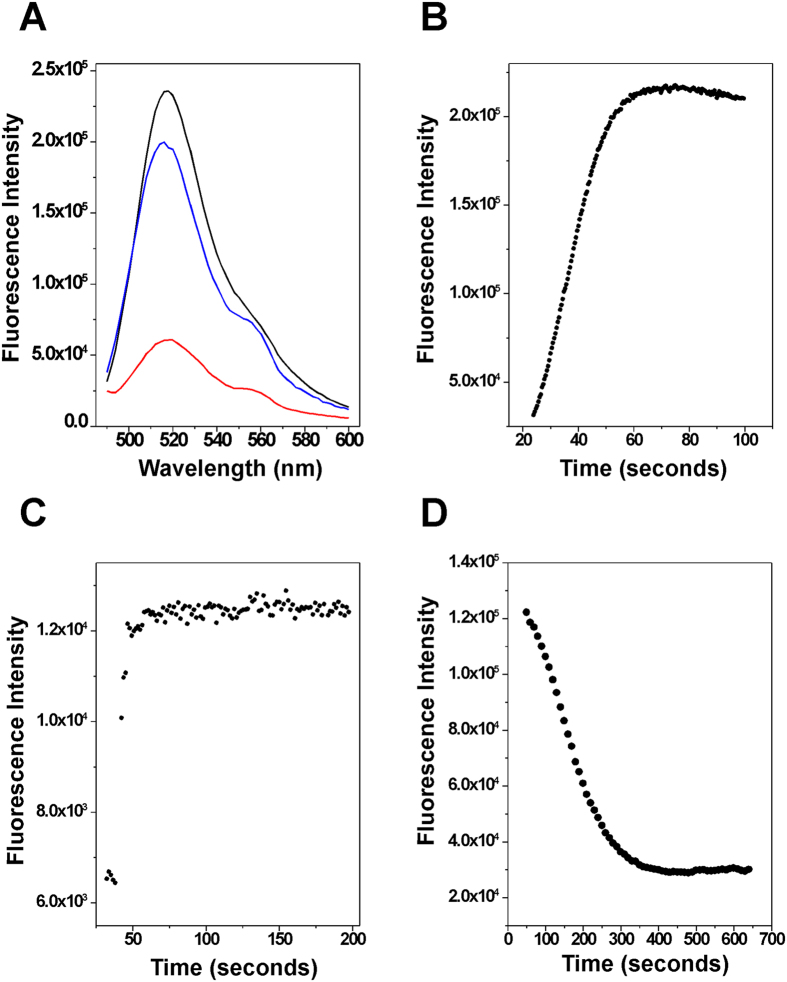
(**A**) Fluorescence spectra of sc (red line), rx (black line), and nk (blue line) pAB1_FL509. λex  = 470 nm. (**B**) Kinetics of the nicking reaction by Nt.BbvCI. Briefly, 60 μL of 1 × CutSmart buffer containing 500 ng of sc pAB1_FL905 was prepared and equilibrated to 37 °C. 20 units of Nt.BbvCI were added to initiate the nicking reaction. The fluorescence intensity at λem = 521 nm was monitor with λex = 470 nm. (**C**) Kinetics of the relaxation reaction by E. coli DNA topoisomerase I. For the relaxation reaction, 90 μL of 1 × NEBuffer 4 (50 mM KAc, 20 mM Tris-Ac, 10 mM Mg(AC)_2_, 1 mM DTT, pH 7.9) containing 270 ng of sc pAB1_FL905 was prepared and equilibrated to 37 °C. 0.67 μM of *E. coli* DNA topoisomerase I was used to relax the sc pAB1_FL905. The fluorescence intensity at λem = 521 nm was monitor with λex = 470 nm. (**D**) Kinetics of the supercoiling reaction by E. coli DNA gyrase. For the supercoiling reaction, 90 μL of 1 × gyrase buffer containing 1 μg of rx pAB1_FL905 was prepared and equilibrated to 37 °C. 30 units of *E. coli* DNA gyrase was used to supercoil the rx pAB1_FL905. The fluorescence intensity at λem = 521 nm was monitor with λex = 470 nm.

After we obtained rx and sc pAB1_FL905, we compared fluorescence properties of sc, rx, and nicked (nk) pAB1_FL905. Figure 3A shows our results. As expected, the fluorescence intensity of rx or nk pAB1_FL905 is significantly higher than that of the sc pAB1_FL905. Figure 3B–D show kinetic results of pAB1_FL905 reacting with three different enzymes: Nt.BbvCI, *E. coli* topoisomerase I, and *E. coli* DNA gyrase. As anticipated, Nt.BbvCI was able to quickly nick sc pAB1_FL905 with a half-life of ~15 seconds (Fig. 3B). This result supports previous conclusions regarding (AT)_n_ that undergoes very rapid cruciform formation, as no detectable kinetic barrier prevents rapid interconversion between extruded and unextruded conformations in sc plasmid DNA templates^10^. It also suggests that pAB1_FL905 is a good DNA substrate to study DNA topology and topoisomerases by FRET. Similarly, large amounts of *E. coli* DNA topoisomerase I was able to rapidly relax sc pAB1_FL905 (Fig. 3C). The kinetics of *E. coli* DNA gyrase was relatively slow (Fig. 3D). Nevertheless, further studies are required to determine kinetic parameters of these enzymes.

We also designed three similar fluorescence-labeled oligomers. FL919 and FL920 contain a dabcyl-labeled dT at 27th and 31^st^ position, and a fluorescein-labeled dT at 57th and 53th position from the 5′-end, respectively. In other words, the distance between the dabcyl and fluorescein is different for FL905, FL919, and FL920. FL924 carries a BHQ2-labeled dT at 29th position from the 5′-end (the 8^th^ position of the AT sequence from the 5′ end; Figure S5) and a TAMRA-labeled dT at 55th position from the 5′-end (the 34^th^ position of the AT sequence from the 5′ end, Figure S5). These three oligomers were inserted between the two Nt.BbvCI sites of pAB1 to yield rx and sc pAB1_FL919, pAB1_FL920, and pAB1_FL924. Figures S4 and S5 show fluorescence properties of these DNA molecules. Similar to pAB1_FL905, the fluorescence intensity of rx pAB1_FL919, pAB1_FL920, and pAB1_FL924 is significantly higher than that of the sc DNA molecules (Figures S4 and S5). However, the FRET efficiency of pAB1_FL919 and pAB1_FL920 is lower than that of pAB1_FL905. This result demonstrated that FL905 has the optimal distance between dabcyl and fluorescein for studying supercoiling-dependent transitions of pAB1 by FRET. As expected, the fluorescence intensity of pAB1_FL924 is lower than that of pAB1_FL905 although the FRET efficiency is similar for both DNA molecules.

### Potential applications

**Figure 4:**
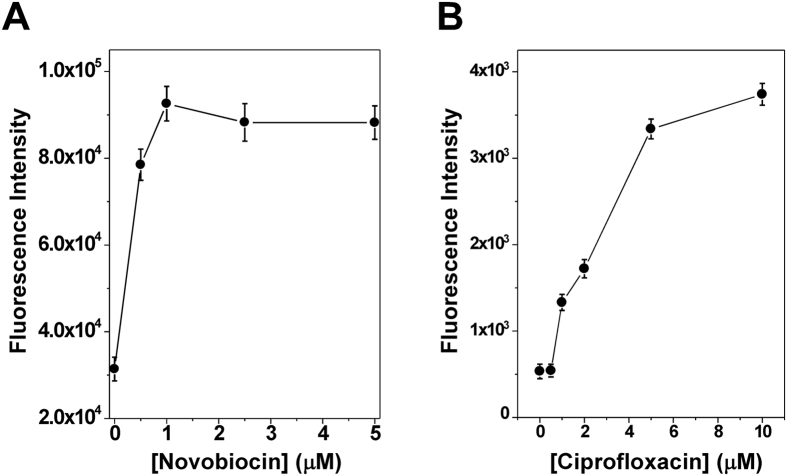
DNA gyrase was potently inhibited by novobiocin (**A**) and ciprofloxacin (**B**). For DNA supercoiling reactions, 60 μL μL of 1 × gyrase buffer containing 670 ng of of rx pAB1_FL905 was prepared and equilibrated to 37 °C. 20 units of DNA gyrase was used to supercoil the rx pAB1_FL905 in the presence of different concentrations of novobiocin and ciprofloxacin. The fluorescence intensity at λem = 521 nm was monitor with λex = 494 nm. The inhibition IC50 was estimated to be 0.48 ± 0.14 and 2.57 ± 1.b μM for novobiocin and ciprofloxacin, respectively.

Rx or sc fluorescently labeled pAB1_FL905 or similar DNA molecules should have many potential applications. As shown above, they can be used to study supercoiling-dependent DNA topological changes or determine biochemical properties and kinetics of various DNA topoisomerases. Neverteless, in our opinion, the most important application of these DNA molecules is to screen inhibitors or compounds targeting different DNA topoisomerases since many of these compounds are either anticancer drugs, such as doxorubicin^19^, or antibiotics, such as ciprofloxacin^20^. Figure S6A shows a strategy for identifying bacterial DNA gyrase inhibitors. In the absence of gyrase inhibitors, bacterial DNA gyrase is capable of converting rx DNA templates into sc DNA molecules. As demonstrated above, this conversion results in quenching of fluorescence of pAB1_FL905. However, DNA gyrase inhibitors should inhibit this conversion. In this way, the fluorescence intensity of rx pAB1_FL905 should not be changed. A titration experiment should also yeild an inhibition IC50 for the gyrase inhibitor. According to this strategy, we performed titration experiments in which different concentrations of novobiocin and ciprofloxacin were added into DNA supercoiling assays. Figure 4 shows our results. Novobiocin and ciprofloxacin potently inhibited the activities of DNA gyrase with an estimated IC50 of 0.48 ± 0.14 and 2.57 ± 1.62 μM, respectively. Agarose gel electrophoresis confirmed that these antibiotics indeed potently inhibited DNA gyrases activities (Figure S6B). Due to simplicity, this FRET assay can be easily to adapt into a high throughput format to identify gyrase inhibitors from millions of compounds found in small molecule libraries. Similar assays may be used to identify inhibitors targeting other DNA topoisomerase, such human DNA topoisomerase I and II. Furthermore, fluorescently labeled sc or rx plasmid DNA molecules may be used to study DNA recombination^16^ or other supercoiling-dependent structural transitions, such as supercoiling-induced G-quadruplex^21^.

In summary, we have invented a novel method to produce a type of fluorescence-labeled rx or sc DNA molecule, such as pAB1_FL905 in the milligram range. This type of DNA molecules could be developed into powerful tools to study DNA topology and topoisomerases. Since only a few nano grams of pAB1_FL905 are needed for 384-well or 1536-well plates for detection, these DNA molecules could be used to develop rapid and efficient high-throughput screening assays to identify inhibitors from the millions of compounds found in small molecule libraries that may target DNA topoisomerases, such as bacterial DNA gyrase and human DNA topoisomerase I & II.

## Methods

### Materials

Restriction enzymes Nt.BbvCI, SphI, BamHI, *E. coli* DNA gyrase, and T4 DNA ligase were purchased from New England Biolabs (Beverly, MA, USA). *E. coli* DNA topoisomerase I was purified as described previously^22^. The following synthetic oligonucleotides were purchased from MWG-Biotech, Inc. (Huntsville, AL): FL882 (5′-CCCTCAGCCCGACAGCACGAGACGATATATATATATATATATATATATATATATATATATATATATGGGCCAACCAACCAGCCCCTCAGCG-3′), FL883 (5′-GATCCGCTGAGGGGCTGGTTGGTTGGCCCATATATATATATATATATATATATATATATATATATATATATCGTCTCGTGCTGTCGGGCTGAGGGCATG-3′), FL905 (5′-TCAGCCCGACAGCACGAGACGATATATA[Dab-dT]ATATATATATATATATATATATATA[Fl-dT]ATATATATGGGCCAACCAACCAGCCCC-3′), FL919 (5′-TCAGCCCGACAGCACGAGACGATATA[Dab-dT]ATATATATATATATATATATATATATATA[Fl-dT]ATATATGGGCCAACCAACCAGCCCC-3′), FL920 (5′-TCAGCCCGACAGCACGAGACGATATATATA[Dab-dT]ATATATATATATATATATATA[Fl-dT]ATATATATATGGGCCAACCAACCAGCCCC-3′), and FL924 (5′-TCAGCCCGACAGCACGAGACGATATATA[BHQ2-dT]ATATATATATATATATATATATATA[TAM-dT]ATATATATGGGCCAACCAACCAGCCCC-3′) where Dab-dT, Fl-dT, BHQ2-dT, and TAM-dT represent dabcyl-dT, fluorescein-dT, BHQ2-dT, and TAMRA-dT, respectively. QIAquick Nucleotide Removal Kit and QIAquick Gel Extraction Kit were obtained from Qiagen, Inc (Valencia, CA).

### Plasmids

Plasmid pAB1 (2,757 bp) was constructed by inserting a 95 bp synthetic DNA fragment FL_AT42 (the annealing product of FL882 and FL883) between the SphI and BamHI sites of pUC18. DNA sequencing was used to verify the inserted DNA sequence.

### Synthesis of relaxed (rx) and supercoiled (sc) pAB1_FL905, pAB1_FL919, pAB1_FL920, and pAB1_FL924

For a typical small scale of reaction, 10 μg of pAB1 (~5.7 pmol) was digested by 25 units of Nt.BbvCI in 200 μL of 1 × CutSmart Buffer (50 mM KAc, 20 mM Tris-Ac, 10 mM Mg(AC)_2_, 100 μg/mL BSA, pH 7.9). After the digestion, 80 pmol of phosphorylated FL905 was added into the reaction mixture. The reaction mixture was incubated at 90 °C in a 4-liter water bath for one minute and then cooled down to room temperature in the water bath (~4 to 5 hours; usually this step was carried out overnight). To generate rx pAB1_FL905, 300 units of T4 DNA ligase were added into the reaction mixtures in the presence of 10 mM of DTT and 2 mM of ATP (final concentrations). The reaction mixtures were incubated at 37 °C to seal the nicks and yield rx pAB1_FL905. The rx pAB1_FL905 was separated by 1% agarose gel electrophoresis and purified by QIAquick Gel Extraction Kit. Typically, we were able to obtain ~6 μg of rx pAB1_FL905 (~60% yield). To produce sc pAB1_FL905, 1 μg of rx pAB1_FL905 was treated with 5 units of *E. coli* DNA gyrase for 1 hour at 37 °C. The sc pAB1_FL905 can be purified by QIAquick Nucleotide Removal Kit or separated by 1% agarose gel and purified by QIAquick Gel Extraction Kit. An alternative procedure was also used to produce sc pAB1_FL905. First, the annealed product of the Nt.BbvCI digested pAB1 and FL905 was purified by QIAquick Nucleotide Removal Kit. The purified DNA sample (~1 μg) was ligated with 300 units of T4 DNA ligase in the presence of 5 units of DNA gyrase. The sc and rx pAB1_FL905 were separated by using a 1% agarose gel and purified by using QIAquick Gel Extraction Kit. Rx and sc pAB1_FL919, pAB1_FL920, and pAB1_FL924 were also generated similarly.

For a typical large scale of reaction, 1 mg of pAB1 (~570 pmol) was digested by 2,500 units of Nt.BbvCI in 20 mL of 1 × CutSmart Buffer for one hour at 37 °C. After the digestion, 8,000 pmol of phosphorylated FL905 was added into the reaction mixture. The reaction mixture was incubated at 90 °C in a 4-liter water bath for two minutes and then cooled down to room temperature in the water bath (~4 to 5 hours; usually this step was carried out overnight). To generate rx pAB1_FL905, 25,000 units of T4 DNA ligase were added into the reaction mixtures in the presence of 10 mM of DTT and 2 mM of ATP (final concentrations). The reaction mixtures were incubated at 37 °C to seal the nicks and yield the relaxed pAB1_FL905. The unpurified rx pAB1_FL905 sample was extracted with 20 mL of phenol, precipitated with ethanol, and washed once with 70% ethanol. Rx pAB1_FL905 was purified by CsCl-EB equilibrium gradient banding. A total of 486 μg of rx pAB1_FL905 was produced by this procedure. To generate sc pAB1_FL905, the ligation reaction was carried out in the presence of 25 μM of ethidium bromide. The unpurified sc pAB1_FL905 sample was extracted twice with 20 mL of phenol, precipitated with ethanol, and washed once with 70% ethanol. Sc pAB1_FL905 was purified by CsCl-EB equilibrium gradient banding. Alternatively, after phenol extraction and ethanol precipitation, the unpurified rx pAB1_FL905 sample was treated with *E. coli* DNA gyrase in the presence of 2 mM of ATP at 37 °C for one hour to yield sc pAB1_FL905. Sc pAB1_FL905 was purified by CsCl-EB equilibrium gradient banding. A total of 571 μg of sc pAB1_FL905 was obtained.

### Fluorescence spectroscopy

Fluorescence measurements were preformed using an ISS, Inc., PC1 photo counting spectrofluorimeter with an excitation wavelength of 470 nm and bandwidth resolution of ±4 nm or a Biotek Synergy H1 Hybrid Plate Reader with an excitation wavelength of 482 nm.

### Gyrase inhibition assay

DNA gyrase inhibition assays were performed in 50 μL of 1× gyrase buffer (35 mM Tris-HCl, 24 mM KCl, 4 mM MgCl_2_, 2 mM DTT, 1.75 mM ATP, 5 mM spermidine, 0.1 mg/mL BSA, 6.5% glycerol, pH7.5) containing 560 ng of of rx pAB1_FL905 and equilibrated to 37 °C. 20 units of DNA gyrase was used to supercoil the rx pAB1_FL905 in the presence of different concentrations of novobiocin and ciprofloxacin. The fluorescence intensity at λ_em_ = 521 nm was monitored with λ_ex_ = 494 nm in a microplate reader. The IC50 values were estimated by nonlinear fitting of the following equation: 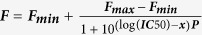 where F is the fluorescence intensity at the x concentration of an inhibitor. F_max_ and F_min_ are the maximum and minimum fluorescence of the DNA sample, respectively. P is a slope parameter.

### Molecular Modeling

DNA molecular models are generated using HyperChem 8.0.

## Additional Information

**How to cite this article**: Gu, M. *et al*. Fluorescently labeled circular DNA molecules for DNA topology and topoisomerases. *Sci. Rep*. **6**, 36006; doi: 10.1038/srep36006 (2016).

**Publisher’s note:** Springer Nature remains neutral with regard to jurisdictional claims in published maps and institutional affiliations.

## Supplementary Material

Supplementary Information
