# Supplementary material for: Fluorescently labeled circular DNA molecules for DNA topology and topoisomerases
Source: Sci Rep. 2016 Oct 31;6:36006. doi: 10.1038/srep36006 (PMC5087112; doi:10.1038/srep36006)
Supplement: Supplementary Information [file srep36006-s1.pdf]

Fluorescently labeled circular DNA molecules for DNA topology and topoisomerases

Maxwell Gu[1, 2, #], Andrea Berrido[1, 2], Walter G. Gonzalez[1, 2], Jaroslava Miksovska[1, 2], Jeremy Chambers[1, 3] and Fenfei Leng[1, 2, *]

[1]Biomolecular Science Institute, [2]Department of Chemistry & Biochemistry, Florida International University, Miami, FL 33199

[3]Department of Cellular Biology and Pharmacology, Herbert Wertheim College of Medicine, Florida International University, Miami, FL 33199

* To whom correspondence should be addressed: Department of Chemistry & Biochemistry, Florida International University, 11200 SW 8th Street, FL 33199. Tel: 305-348-3277; Fax: 305-348-3772; E-mail: lengf@fiu.edu

[#]Maxwell Gu is a high school student at American Heritage School, Plantation, FL 33325

Supplemental Information

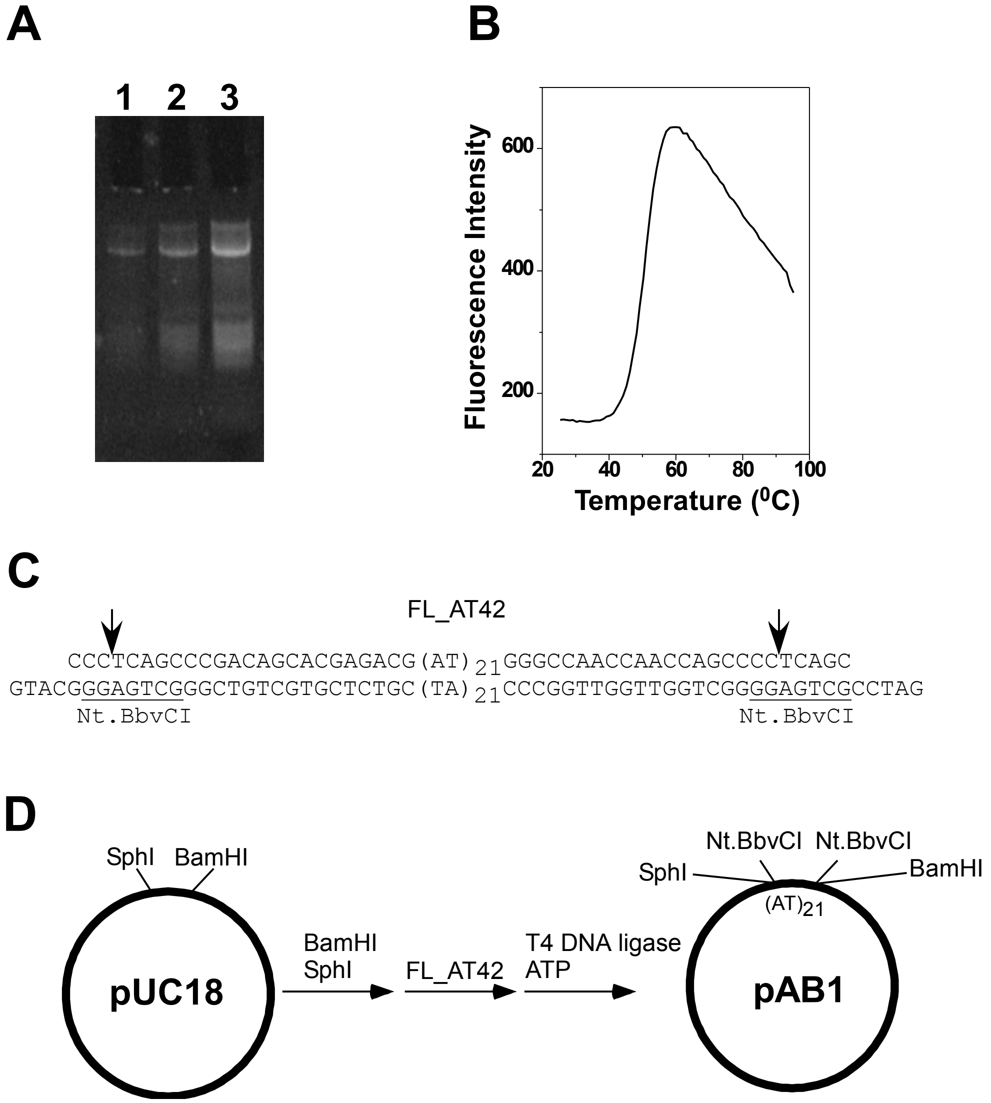

Figure S1. (A) 20% polyacrylamide gel of FL905 in 1×TAE without staining. **(B)** Fluorescence DNA melting of 1 μM of FL905 in 10 mM Tris-HCl, pH 8, 50 mM NaCl with λ_{ex} = 494 nm and λ_{em} = 521 nm. **(C)** The double-stranded oligomer FL_AT42 carrying a 42 bp AT sequence that was inserted between SphI and BamHI sites of pUC18. FL_AT42 also contains two Nt.BbvCI recognition sites on the same orientation. Arrows indicate the nicking sites. **(D)** The cloning steps to insert FL_AT42 into SpHI and BamHI sites to yield pAB1 that contains two Nt.BbvCI sites. The 42 bp AT sequence is located between the two Nt.BbvCI sites.

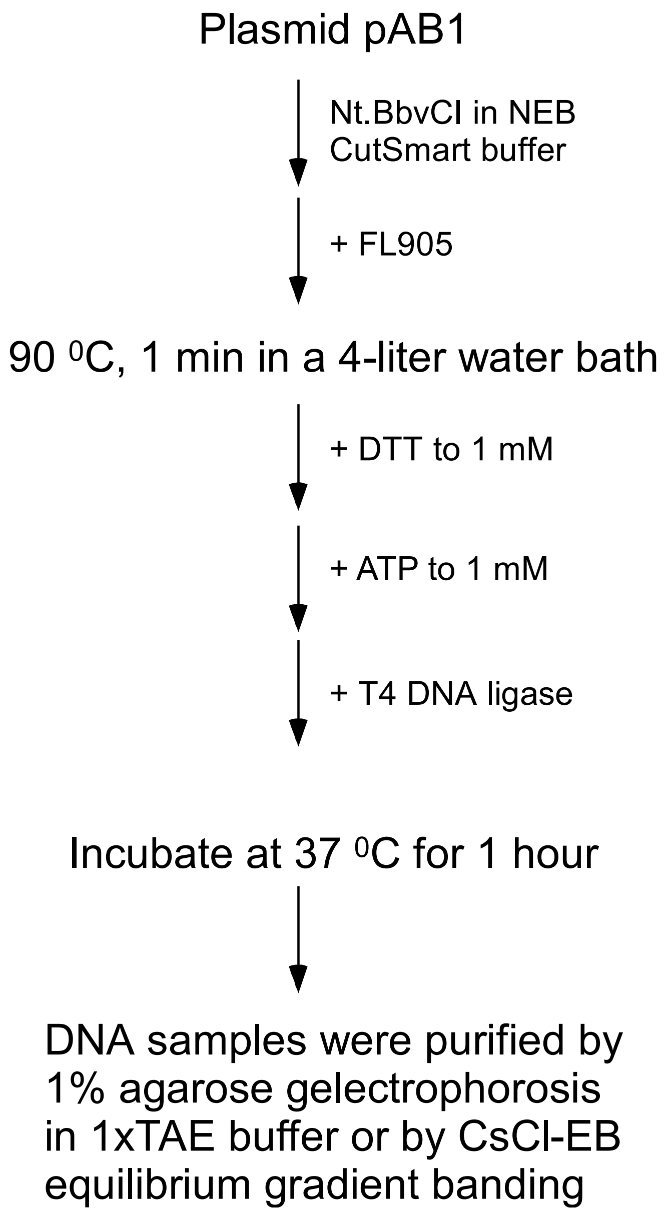

Figure S2. An experimental procedure to produce relaxed pAB1_FL905. The supercoiled pAB1_FL905 can be generated by the treatment of the relaxed pAB1_FL905 with *E. coli* DNA gyrase in the presence of ATP.

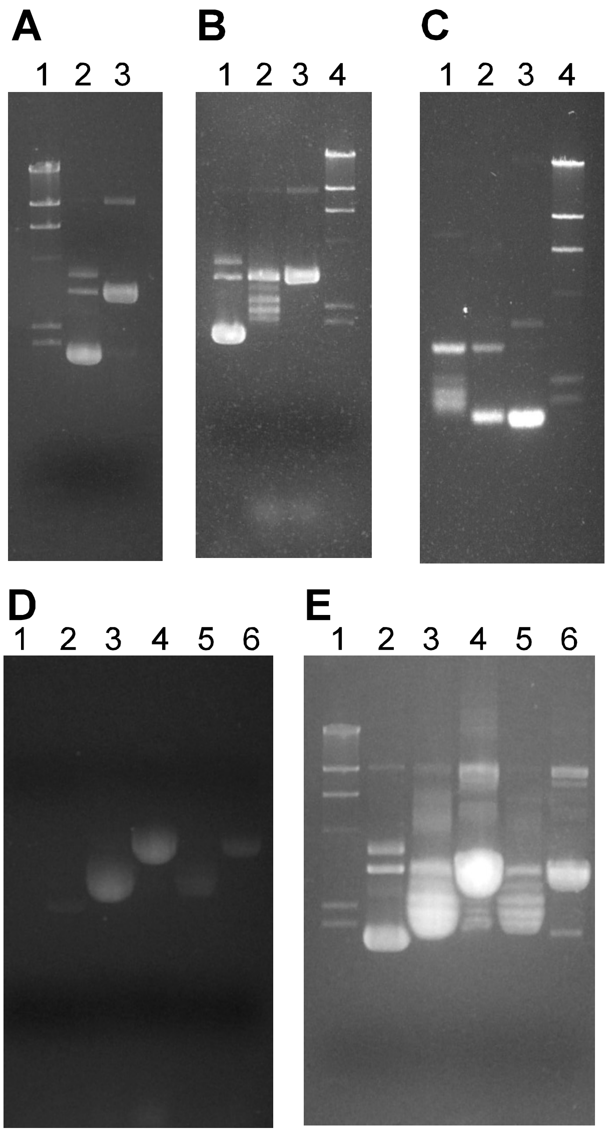

Figure S3. 1% agarose gels to analyze pAB1 and pAB1_FL905. (A) Digestion of pAB1 by Nt.BbvCI. Lane 1 is lambda DNA HindIII digest. Lanes 2 and 3 are pAB1 before and after Nt.BbvCI digestion. **(B)** The annealed pAB1 with FL905 before (lane 3) and after (lane 2) the ligation reaction by T4 DNA ligase. The DNA sample is slightly (+) supercoiled because the ligation reaction was performed at 37 °C and the gel was run at 24 °C. Lane 1 is undigested pAB1 and lane 4 is lambda DNA HindIII digest. **(C)** DNA supercoiling assays to convert the relaxed pAB1_FL905 (lane 1) into supercoiled pAB1_FL905 (lane 2) that was purified by QIAquick Gel Extraction Kit. Lane 3 is undigested pAB1 and lane 4 is lambda DNA HindIII digest. **(D)** and **(E)** show the DNA samples after the relaxed pAB1_FL905 was purified by CsCl-EB gradient banding. **(D)** is the image of the gel before EB staining and the bands represent the intrinsic fluorescence of fluorescein. Lane 2 is sc pAB1_FL905 and lane 1 is lambda DNA HindIII digest. Lanes 3 and 5 are re pAB1_FL905. Lanes 4 and 6 are nk pAB1_FL905.

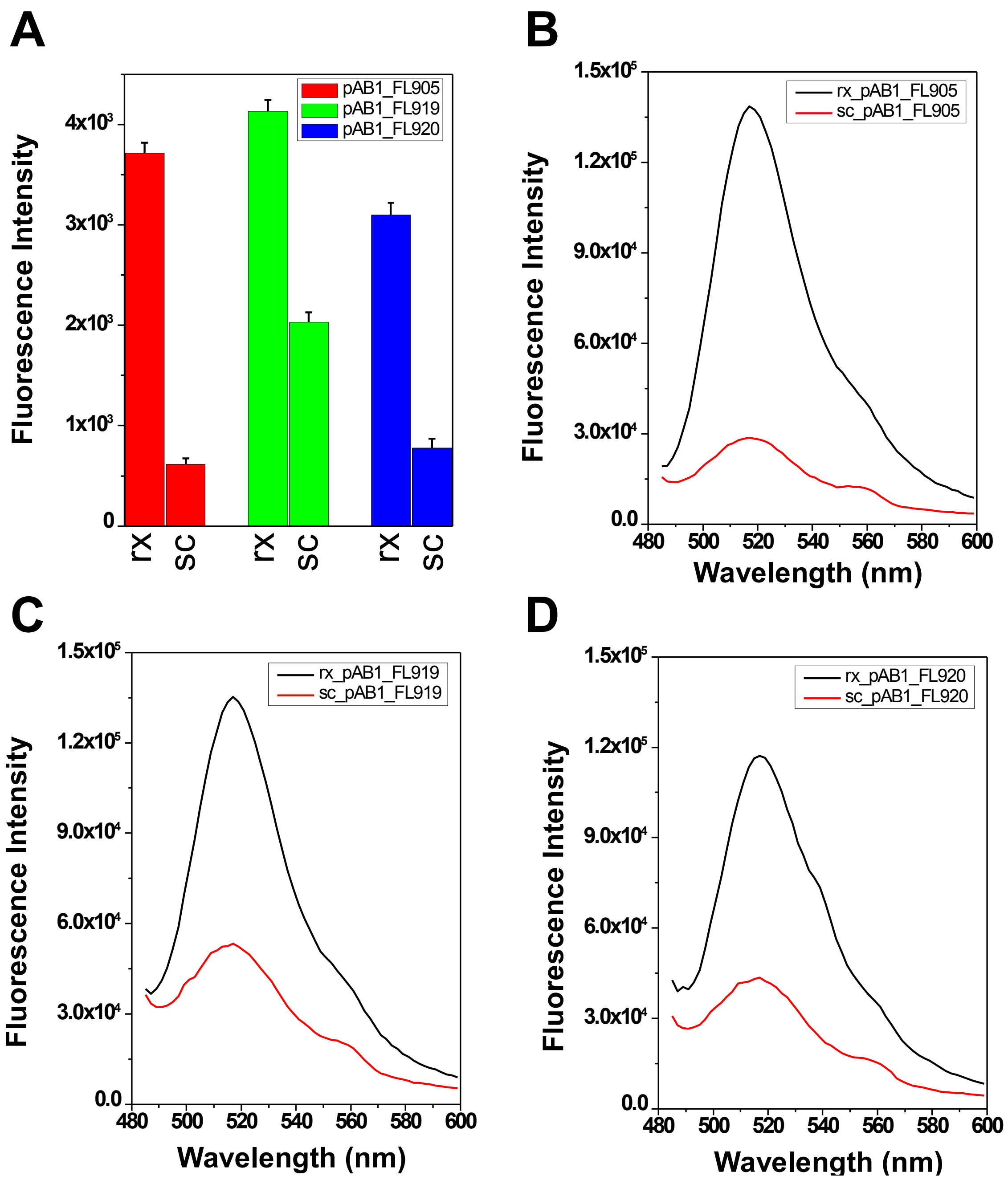

Figure S4. (A). Fluorescence intensity of rx and sc pAB1_FL905, pAB1_FL919, and pAB1_FL920. The fluorescence intensity was measured by a plate reader with λ_{ex} = 494 nm and λ_{em} = 521 nm. The FRET efficiency was estimated to be 0.83, 0.51 and 0.75 for pAB1_FL905, pAB1_FL919, and pAB1_FL920, respectively. The FRET efficiency (E) was calculated by the following equation: $E = 1 - \frac{I_{sc}}{I_{rx}}$ where I_{sc} and I_{rx} represent the fluorescence intensity of supercouled and relaxed DNA molecules, respectively.**(B)**, **(C)**, and **(D)** show fluorescence spectra of sc (red lines) and rx (black lines) pAB1_FL905, pAB1_FL919, and pAB1_FL920, respectively. λ_{ex} = 470 nm.

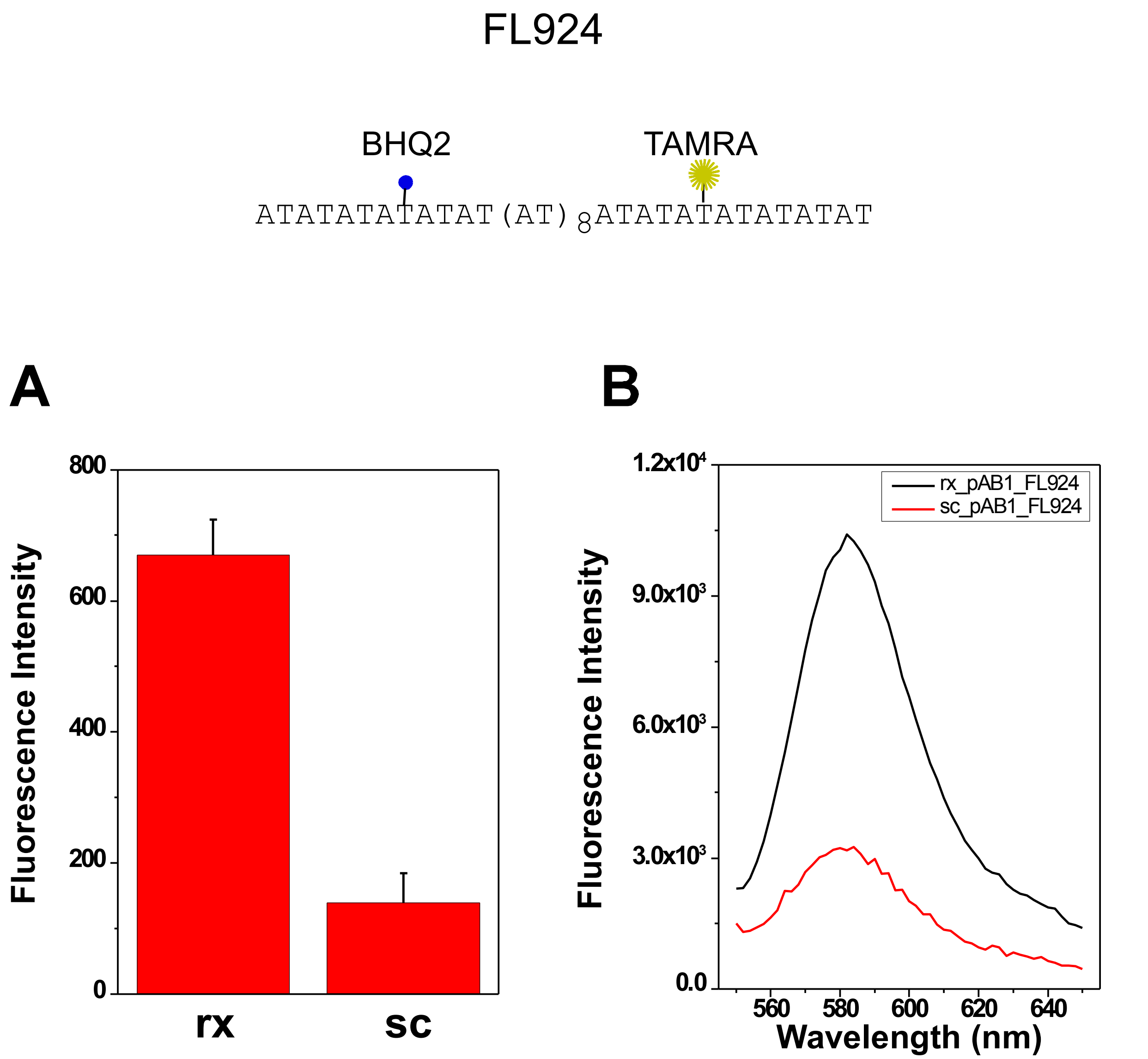

Figure S5. **(A)** Fluorescence intensity of rx and sc pAB1_FL924 that contains oligomer FL924. The fluorescence intensity was measured by a plate reader with λ_{ex} = 550 nm and λ_{em} = 580 nm. The FRET efficiency was estimated to be 0.80. **(B)** Fluorescence spectra of sc (red lines) and rx (black lines) pAB1_FL924 with λ_{ex} at 550 nm.

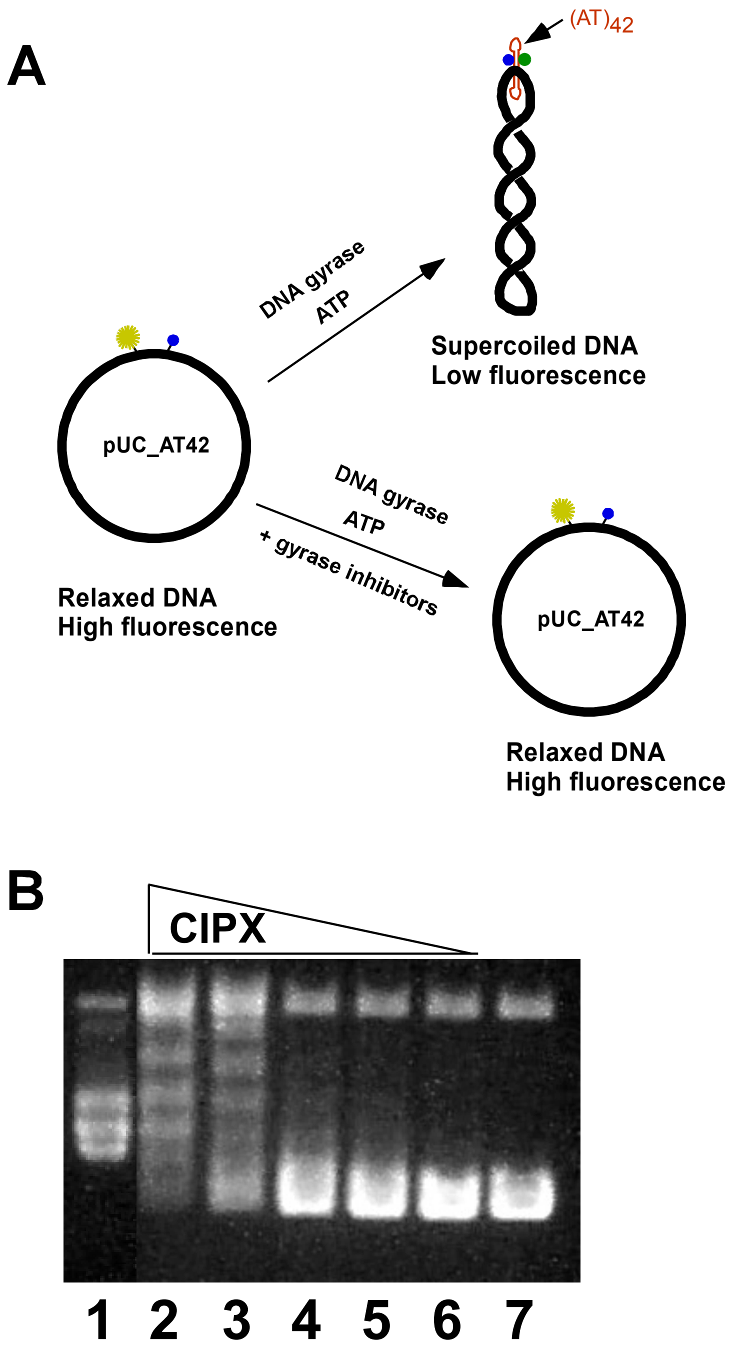

Figure S6. (A) An experimental strategy to screen inhibitors targeting bacterial DNA gyrase. **(B)** DNA gyrase was inhibited by ciprofloxacin monitored by 1% agaorose gel electrophoresis. Briefly, 60 μL of 1×gyrase buffer containing 670 ng of of rx pAB1_FL905 was prepared and equilibrated to 37 °C. 20 units of DNA gyrase was used to supercoil the rx pAB1_FL905 in the absence (lane 1) or presence of different concentrations of ciprofloxacin. The supercoiling reactions were terminated by addition of EDTA to 20 mM followed by phenol extraction. The DNA samples were loaded into a 1% agarose gel in 1×TAE buffer to resolve DNA topoisomers. Lanes 2 to 6 contained 10, 8, 4, 2, and 1 μM of ciprofloxacin. Lane 7 contains sc pAB1_FL905.
